# Changing Trends in the Prevalence, Seasonal Distribution and Antimicrobial Susceptibility Pattern of Salmonella: An 11-Year Retrospective Study at a Tertiary Care Hospital

**DOI:** 10.7759/cureus.102364

**Published:** 2026-01-27

**Authors:** Anjum Mir, Uksim Qadri, Shugufta Roohi, Tabish Qayoom, Iram Jan, Sabah Rashid Bhat, Shaista Nazir Baba, Bashir A Fomda

**Affiliations:** 1 Microbiology, Sher-i-Kashmir Institute of Medical Sciences, Srinagar, IND

**Keywords:** antimicrobial resistance, prevalence, salmonella paratyphi a, salmonella paratyphi b, salmonella typhi, salmonellosis, seasonal variation

## Abstract

Objectives

Salmonella causes enteric fever, a major public health issue in India. The emergence of drug-resistant strains is concerning. This study assessed the prevalence, seasonal distribution, and antibiogram of Salmonella from blood cultures at a tertiary care hospital in North India.

Materials and methods

This retrospective descriptive study was conducted in the Department of Microbiology at a tertiary care centre over 11 years. Blood samples received in the laboratory were processed using the BacT/ALERT system. Identification and antimicrobial susceptibility testing were performed with VITEK 2 (bioMérieux, France). *Escherichia coli* ATCC 25922 and *Pseudomonas aeruginosa* ATCC 27853 were used as control strains.

Results

Of 67,085 blood cultures, 26,796 (39.9%) were positive, yielding 761 Salmonella isolates: S. Typhi (578, 75.9%), S. Paratyphi A (179, 23.5%), and S. Paratyphi B (4, 0.6%). Outpatients contributed 377 (50.5%) isolates. Prevalence peaked in summer. All isolates showed high susceptibility to ceftriaxone (99.1%), cotrimoxazole (99.2%), and ampicillin (91.8%). Ciprofloxacin susceptibility declined from 25% (2014) to 5% (2024), and levofloxacin from 20% to 10%.

Statistical analysis

Data were analyzed using SPSS version 23.0 (IBM Corp., Armonk, NY, USA). Categorical variables were expressed as frequencies and percentages, with appropriate tables for data presentation.

Conclusions

This study revealed that *Salmonella *Typhi was the most common species isolated, and a high prevalence of salmonellosis was observed during the summer season. Also, an increased resistance among *Salmonella *isolates to fluoroquinolones was seen, whereas the sensitivity to ceftriaxone was excellent.

## Introduction

Salmonella is a Gram-negative, flagellated, capsulated bacillus first isolated by an American bacteriologist, D. E. Salmon from porcine intestine in 1884 [1]. Kauffmann proposed a concept from which the nomenclature of genus Salmonella has evolved based on the serologic identification of O (somatic) and H (flagellar) antigens according to which each serotype was considered a separate species. Other taxonomic proposals have been based on the strain's clinical role and the biochemical traits classifying serotypes into subgenera [2]. It is responsible for causing enteric fever that is prevalent worldwide, more so in low-income and middle-income countries (LMICs), and is used as a combined name for typhoid fever and paratyphoid fever. It is transmitted through contaminated water or food. The peak incidence of Salmonella Typhi infection occurs between the months of April and June (dry season), followed by July and September (monsoon season) in India. Both these diseases have a similar clinical presentation but exhibit different propensities to develop resistance to antimicrobials [3,4]. The mainstay of diagnosing typhoid fever is a positive blood culture, the sensitivity of which is 40-60% and is usually beneficial in the early course of the disease. The sensitivity of stool and urine cultures is much lower. Bone marrow cultures are more sensitive but are difficult to obtain, relatively invasive, and of little use in public health settings [5]. The antimicrobial resistance of Salmonella is growing and the main contributing factors for the same are over-prescription and indiscriminate use of antibiotics [4].

The standard treatment for typhoid fever in the 1940s was chloramphenicol. There was an emergence of reports of treatment failure and outbreaks of chloramphenicol-resistant typhoid reported from India, Vietnam, Peru, and Korea in the 1970s. By this time, ampicillin and trimethoprim-sulfamethoxazole were included as the first-line antibiotics used to treat enteric fever. However, a large outbreak of multidrug-resistant (MDR) strains resistant to chloramphenicol, ampicillin and cotrimoxazole occurred in Mexico in 1972. In India, in 1980, the MDR-associated H58 haplotype of S. Typhi emerged, which is now responsible for the majority of new typhoid fever cases in South Asia, East Africa, and parts of Southeast Asia. In the 1990s, fluoroquinolones became the recommended treatment option but soon after, fluoroquinolone nonsusceptibility (FQNS) emerged, which is now found across South Asia and is rapidly increasing in parts of Africa and Southeast Asia. Pakistan has experienced an outbreak of typhoid caused by extensively drug-resistant (XDR) since 2016. Azithromycin and carbapenems are the only remaining effective treatments for XDR S. Typhi. However, Azithromycin-resistant typhoid has independently emerged in multiple countries [6]. The first-ever report of multipledrug-resistant Salmonella Typhi epidemic from Asia was reported in April 1988 in Baramulla town of Kashmir, wherein a very rare multiple drug-resistant strain of S. typhi was identified as phage type biotype II untypeable (UVS2) [7]. In an observational study conducted over three years at our tertiary care hospital, all Salmonella isolates were resistant to chloramphenicol and cloxacillin but sensitive to ceftriaxone and ciprofloxacin [8]. Apart from previous studies on Salmonella, there is limited data on various aspects of salmonellosis from this region. This study thus aims to determine the prevalence of Salmonella spp. in bloodstream infections, its seasonal distribution, and the antimicrobial susceptibility pattern among patients with febrile illness visiting a tertiary care hospital.

## Materials and methods

Study design and setting

This 11-year retrospective descriptive study was carried out in the Department of Microbiology at Sher-i-Kashmir Institute of Medical Sciences, which is a superspeciality tertiary care teaching hospital. The hospital caters to a large population, receiving referrals from both rural and urban areas, thereby ensuring a wide representation of patient samples. The study was conducted from January 2013 to August 2024. This study period was chosen to capture long-term temporal trends in the prevalence, seasonal distribution, and antimicrobial susceptibility patterns of Salmonella spp. over more than a decade. This extended duration allowed assessment of year-to-year variability, seasonal fluctuations, and evolving antimicrobial resistance patterns. Additionally, the period coincides with the availability of consistent automated blood culture and susceptibility testing systems (BacT/ALERT® and VITEK® 2) in our laboratory, ensuring methodological uniformity and reliable longitudinal comparisons.

Inclusion and exclusion criteria

Inclusion: All blood culture samples received in the bacteriology section of the laboratory during the study period were included.

Exclusion: Duplicate or repeat blood culture samples obtained from the same patient during a single clinical episode or hospital admission were excluded to avoid overrepresentation of persistent bacteremia. Only the first positive blood culture isolate per patient per admission was included for analysis. In cases where multiple blood cultures were collected from the same patient within the same episode and yielded Salmonella spp., subsequent isolates were excluded irrespective of their antimicrobial susceptibility profile.

Ethical statement

The study protocol was reviewed and approved by the Institutional Ethics Committee (IEC) of the Sher-i-Kashmir Institute of Medical Sciences (SKIMS), Srinagar, with a waiver of informed consent, under protocol number 395/2025, as the study involved retrospective analysis of anonymised laboratory data with no direct patient contact or intervention.

Sample collection and transport

Blood samples were collected from patients with clinical suspicion of septicemia or bloodstream infection. A strict aseptic technique was employed to minimise contamination. Skin antisepsis was achieved by scrubbing with 70% isopropyl alcohol, followed by application of 2% chlorhexidine or povidone-iodine solution. Blood was collected using a sterile syringe and needle or directly into vacuum blood collection systems. The recommended volume of blood (Adults: 8-10 mL per bottle, Children: 1-5 mL per bottle) was inoculated into aerobic BacT/ALERT® culture bottles (bioMérieux, France) immediately after collection. All inoculated bottles were promptly transported to the laboratory to ensure optimal recovery of pathogens.

Automated blood culture monitoring

In the laboratory, blood culture bottles were loaded into the BacT/ALERT® 3D automated blood culture system and incubated at 35 ± 2°C. The system continuously monitored the bottles using a colorimetric sensor technology that detects CO₂ production from microbial metabolism, with readings taken every 10 minutes. Bottles that flagged positive were removed immediately for processing. Bottles remaining negative were incubated for a maximum of five days before being reported as sterile.

Initial processing of positive cultures

Positive bottles were subjected to Gram staining. It was performed directly from the broth, and the preliminary results were communicated promptly to the treating clinician to guide empirical antimicrobial therapy. Simultaneously, subculture was performed by inoculating broth onto 5% sheep blood agar (Hi-Media, India) and MacConkey agar (Hi-Media, India). Culture plates were incubated aerobically at 37°C for 18-24 hours and examined for bacterial growth.

Isolation and preliminary identification

Lactose-fermenting vs. non-lactose-fermenting colonies were differentiated on MacConkey agar. Non-lactose-fermenting colonies were carefully selected for detailed identification. A bacterial suspension was prepared in 0.45% sterile NaCl solution and adjusted to 0.5 McFarland turbidity standard using the VITEK® DensiChek (bioMérieux, France).

Automated identification and antimicrobial susceptibility testing (AST)

The standardized inoculum was inoculated into VITEK® 2 Compact system (bioMérieux, France) for automated processing. Identification (ID) was performed using VITEK® 2 ID-GNB cards, which contain multiple biochemical tests enabling species-level identification. Antimicrobial Susceptibility Testing (AST) was conducted using AST-NO09 cards, covering a broad panel of antimicrobials relevant for Gram-negative pathogens (β-lactams, aminoglycosides, fluoroquinolones, carbapenems, colistin, etc.). Results were expressed as Minimum Inhibitory Concentration (MIC) values, which were automatically categorized as Susceptible (S), Intermediate (I), or Resistant (R) according to the Clinical and Laboratory Standards Institute (CLSI) guidelines applicable for the respective year of testing [9].

Quality control (QC) procedures

To ensure the reliability and reproducibility of both identification and AST results, standard ATCC quality control strains were used throughout the study:

Escherichia coli ATCC 25922 (QC for general Gram-negative susceptibility testing)

Pseudomonas aeruginosa ATCC 27853 (QC for non-fermenters and anti-pseudomonal agents)

These strains were tested at regular intervals in parallel with clinical isolates. All results were reviewed to confirm that QC ranges fell within the acceptable CLSI reference limits.

Data handling and analysis

Demographic and clinical details of patients were obtained from laboratory requisition forms. Microbiological data, including species identification, susceptibility profiles, and resistance patterns, were extracted from the VITEK system and entered into a structured database. Repeat isolates from the same patient were carefully filtered to avoid duplication.

Statistical analysis

Data were analyzed using SPSS version 23.0 (IBM Corp., Armonk, NY, USA). Descriptive statistical methods were employed to summarize the dataset, including calculation of absolute frequencies, proportions, and percentages. Categorical variables such as gender distribution, patient location (OPD/IPD), Salmonella serovar distribution, year-wise and seasonal trends, and antimicrobial susceptibility profiles were summarized as frequencies and percentages. Temporal trends in antimicrobial susceptibility were assessed descriptively across the study years and illustrated using tables and figures. No inferential statistical tests were applied, as the study was descriptive and observational in nature.

## Results

**Figure 1:**
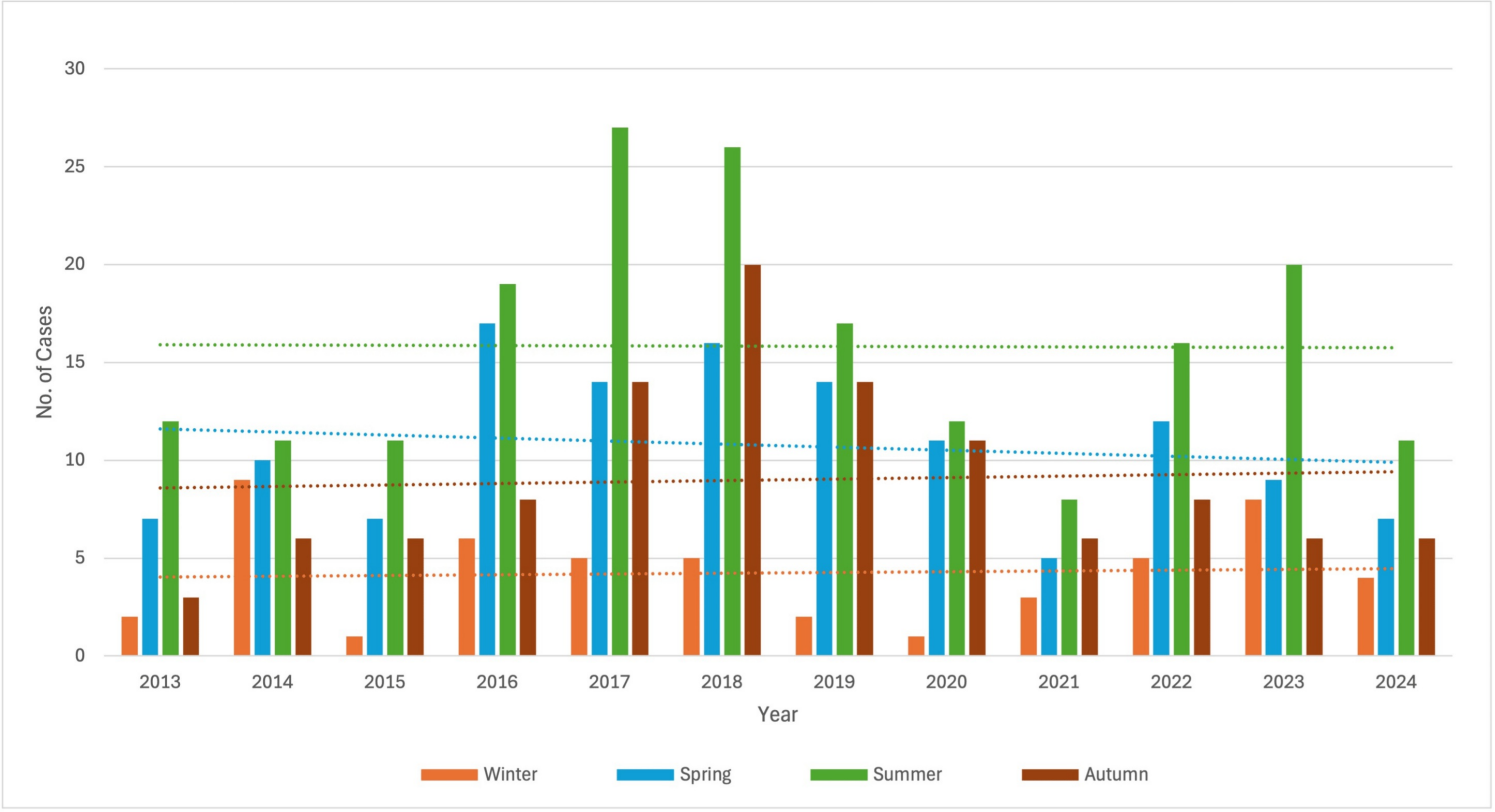
Year-wise and seasonal distribution of Salmonella spp. This clustered bar chart illustrates the number of reported cases stratified by season over 11 years (2013–2024). The y-axis indicates the number of cases, while the x-axis represents calendar years. Each vertical bar corresponds to seasonal case counts for the respective year. Dotted horizontal lines of corresponding colours denote the mean case count for each season across the entire study period, allowing comparison of annual variations against seasonal averages. The figure highlights temporal and seasonal trends, showing higher case burden during summer months with peak counts observed in 2017 and 2018, followed by a gradual decline after 2019.

**Figure 2:**
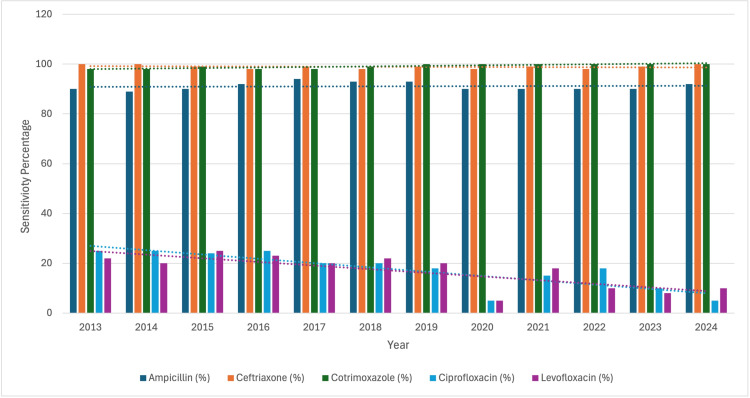
Temporal trends in antimicrobial susceptibility of isolates This clustered bar chart displays the annual antimicrobial susceptibility percentages of isolates tested against five commonly used antibiotics. The y-axis represents the percentage of isolates sensitive to each drug, while the x-axis denotes the study years (2013–2024). Dotted horizontal trendlines indicate the overall direction of sensitivity for each antibiotic across the study period.

During the study period from January 2013 to August 2024, a total of 67,085 blood cultures were processed in the Department of Microbiology. Among these, 26,796 cultures (39.9%) tested positive, with 761 isolates identified as Salmonella. The majority of samples, i.e., n=425 (55.9%), from which Salmonella spp. was isolated, belonged to males, whereas females accounted for n=336 (44.1%) of the total samples. A total of 384 (50.5%) of samples were received from the outpatient department (OPD) and 377 (49.5%) from the inpatient department (IPD). Of these, Salmonella Typhi accounted for the majority (n=578, 75.9%), followed by Salmonella Paratyphi A (n=179, 23.5%) and Salmonella Paratyphi B (n=4, 0.6%) (Table 1). Significantly less number of cases were seen during 2020 due to the COVID-19 pandemic. The prevalence was found to be highest during the summer season for the majority of the study period. A significant increase in the number of cases was observed in 2018, particularly in spring, followed by summer and autumn (Figure 1). All the isolates of Salmonella spp. were highly susceptible to ceftriaxone (n=734, 99.12%), cotrimoxazole (n=735, 99.2%) and ampicillin (n=80, 91.8%). The susceptibility percentage for ciprofloxacin and levofloxacin decreased from 25% in 2014 to 5% in 2024 and from 20% in 2014 to 10% in 2024, respectively (Figure 2).

**Table 1:** Demographic details and distribution of Salmonella spp. This table summarizes the demographic characteristics and distribution of Salmonella isolates obtained from blood cultures processed in the Department of Microbiology during the study period.

Gender	N (%)
Male	425 (55.9%)
Female	336 (44.1%)
Location	
OPD	384 (50.5%)
IPD	377 (49.5%)
Organism	
Salmonella Typhi	578 (75.9%)
Salmonella Paratyphi A	179 (23.5%)
Salmonella Paratyphi B	4 (0.6%)
Total	761 (100%)

## Discussion

This study presents an 11-year retrospective analysis of bloodstream infections caused by Salmonella spp. at a tertiary care centre in North India, focusing on their prevalence, seasonal distribution, and antimicrobial susceptibility patterns. The study highlights the burden of enteric fever in the region and provides insight into the epidemiological trends of Salmonella Typhi and Paratyphi infections. The findings contribute valuable regional data, particularly given the limited published literature from this part of the country. In addition to mapping temporal patterns and demographic distribution, the study evaluates long-term trends in antimicrobial susceptibility, offering a clearer understanding of the shifting resistance landscape and its potential implications for empirical therapy and public health interventions.

Enteric fever is an important cause of morbidity and mortality in Kashmir, and factors contributing to the same include negligible typhoid vaccination coverage and indiscriminate use of antimicrobials [7]. During the current study, 67,085 blood cultures were processed, with 26,796 (39.9%) testing positive, including 761 Salmonella spp. isolates. The overall prevalence of Salmonella spp. was found to be 2.83%. Bhumbla et al., in their study, found the prevalence to be 9.1%, whereas Labi et al. reported the prevalence of Salmonella bacteraemia as 6.5% [10,11]. The variation in prevalence of Salmonella bloodstream infections observed across different studies may be attributed to several epidemiological and methodological factors. Differences in geographic location and endemicity, study design, and population characteristics (hospital-based versus community-based cohorts) significantly influence reported prevalence rates. Variations in healthcare-seeking behavior, blood culture collection practices, and diagnostic yield of culture systems may further contribute to these discrepancies. In addition, regional differences in sanitation standards, access to safe drinking water, and patterns of antimicrobial use are known to affect the transmission dynamics and burden of Salmonella infections. These factors likely explain the lower prevalence observed in the present study compared with higher rates reported in other regions. In a study carried out in Doda district of Jammu and Kashmir, a total of 56.7% healthy people showed positive results that they had typhoid fever based on a clinical examination and serological test [12]. The significant burden of typhoid fever is largely due to the high prevalence of risk factors, including contaminated drinking water, inadequate sanitation, and poor hand hygiene.

Salmonella Typhi was predominant (75.9%), mostly from male patients and OPD samples. A peak in cases was observed in 2018, with seasonal variation, and a decline in 2020 due to COVID-19. Ceftriaxone, cotrimoxazole, and ampicillin showed high susceptibility (>91%), while ciprofloxacin and levofloxacin resistance increased over time. Cephalosporin resistance in S. Typhi was first reported in Bangladesh in 1998 [13]. A major concern arose with the 2016 outbreak of extensively drug-resistant (XDR) typhoid in Sindh, Pakistan, which has since been linked to travel-related cases in countries like the UK and the USA. In India, only a few reports of ceftriaxone-resistant S. Typhi exist, some without and some with genotypic analysis [13].

In the present study, it was observed that a larger number of males than females were positive. These results were in concordance with those in a study by Kalsoom et al. [14]. The higher proportion of males affected in the present study may be explained by increased natural exposure to Salmonella Typhi. In endemic settings, males are more likely to be exposed to contaminated food and water due to greater outdoor activity, occupational exposure, frequent travel, and higher consumption of food from street vendors or workplace canteens. In addition, males often have increased social mobility and participation in communal eating practices, which may raise the risk of ingestion of contaminated food or water. These behavioral and environmental factors, combined with differences in healthcare-seeking patterns, may contribute to the observed male predominance in enteric fever cases. Also, sex-linked differences in the inflammatory response of the host induced by S. Typhi at Peyer’s patches play a role in the higher incidence of typhoid fever in males [15,16].

The prevalence remained the same over the study period. However, significantly less number of cases were isolated during 2020 owing to the COVID-19 pandemic. During the COVID-19 pandemic, there were restrictions for public and private gatherings where food and drinks are normally served, resulting in exposure to Salmonella, such as receptions, parties, festivals, etc. In addition to this, an alteration in healthcare-seeking behaviour, testing policy, diagnostic capacity and reporting compliance also led to the decrease in salmonellosis [17].

In the current study, 50.5% of the cases were from OPD, and these results are consistent with those reported by Saha et al., who, in their study, found that among culture-confirmed enteric fever cases, the majority, i.e. 59%, were OPD cases [16]. This could be due to the fact that the majority of patients were in a stable condition and thus sought care at the OPD, where they were sent home after the prescription of the treatment.

In the current study, out of the total Salmonella spp. isolated, the majority (75.9%) were Salmonella Typhi, followed by Salmonella Paratyphi A and B (23.5% and 0.6% each). The results were comparable to those in a study by Joshi and Yousuf et al., in which they found that 76.6% and 82.2% of Salmonella spp. isolated were Salmonella Typhi, respectively, followed by Salmonella Paratyphi A [18,19]. Patil and Mule, in their study, found 76.5% of Salmonella spp. isolated were Salmonella typhi and 23.5% were Salmonella Paratyphi A [20]. In another study by Gupta et al., 70%-80% and 20%-30% of the isolates were found to be Salmonella Typhi and Salmonella Paratyphi, respectively [21]. The reason for serovar variation could be the smaller inoculum required by Salmonella typhi as compared to Salmonella Paratyphi, which requires a larger inoculum [22]. Also, the significantly lower prevalence of paratyphoid among patients tested in the hospital may be attributed to the fact that paratyphoid presents with mild clinical characteristics and shorter duration of fever as compared to typhoid fever [23].

The maximum number of cases were observed during the summer season. He et al. reported that Salmonella infection mainly occurred seasonally from May to October, that is, the months that are the hottest [24]. In a study carried out by Gorski et al., where the prevalence and serovar diversity of Salmonella enterica were measured during a 5-year survey of surface waters, it was found that it was maximum in spring than in fall [25]. Bacterial pathogens responsible for causing gastrointestinal infection are known to grow more in ambient temperature, which enables their rapid replication. Also, higher concentrations of Salmonella are seen in the food supply in the warmer months owing to its better growth at higher temperatures. Moreover, inadequate cooking practices are also more common during these months because of frequent picnics and barbecue outings [26].

The antimicrobial susceptibility pattern was almost the same throughout the study period. There was a decrease in the susceptibility to quinolones, that is, ciprofloxacin and levofloxacin. A study analysing the year-wise trends in antibiotic resistance profile of S. typhi from 2012 to 2018 revealed a sharp rise in resistance rates of most antibiotics during 2016 and 2017. In contrast to the results of our study, a significant decrease in ciprofloxacin resistance rate and a slight rise in cephalosporin resistance rate among S. typhi isolates from Pakistan were observed in a study carried out by Umair et al. [27]. Ceftriaxone appears to be the most viable treatment agent [28]. However, in a study carried out by Maharjan et al., 95% of the isolates were found to be susceptible to ceftriaxone, and very low sensitivity was observed towards levofloxacin, that is, 20%. All of the isolates were susceptible to cotrimoxazole [29]. The decreasing susceptibility to fluoroquinolone in our study can be attributed to their overuse, as these are the drugs of choice against enteric fever in LMICs because of being cost-efficient, accessible and available in oral formulations. In another study carried out by Varghese et al., an increase in resistance to fluoroquinolones, complete sensitivity to ceftriaxone and co-trimoxazole sensitivity was observed [30].

Limitations

This study was conducted at a single tertiary care center, which may limit the generalizability of findings to the wider community. Antibiotic susceptibility testing was performed only for the antimicrobials included in the available VITEK 2 panels during the study period; therefore, agents such as azithromycin and carbapenems were not consistently evaluated. Being a retrospective study, detailed clinical data such as prior antibiotic exposure, vaccination status, travel history, comorbidities, and treatment outcomes were not available, limiting clinicomicrobiological correlation. Molecular typing and resistance mechanism analysis could not be performed, which would have provided greater insight into the epidemiology of antimicrobial resistance.

## Conclusions

This 11-year retrospective study demonstrates that Salmonella Typhi remains the predominant cause of enteric fever in our region, with a clear seasonal peak during the summer months. While susceptibility to third-generation cephalosporins, particularly ceftriaxone, remains high, a sustained and progressive decline in fluoroquinolone susceptibility was observed over the study period, limiting their utility for empirical therapy. Based on these findings, we recommend that national and regional treatment guidelines be periodically revised using locally generated antimicrobial resistance data. Empirical use of fluoroquinolones for suspected enteric fever should be discouraged in favour of evidence-based alternatives. Strengthening laboratory-based antimicrobial resistance surveillance networks and integrating hospital antibiogram data into public health decision-making should be prioritised. Additionally, rational antibiotic use policies and antimicrobial stewardship programs must be reinforced to prevent further emergence of resistant Salmonella strains.

From a public health perspective, these findings highlight the urgent need for improved preventive strategies, including expansion of typhoid vaccination coverage, enhancement of water sanitation and hygiene infrastructure, and community-level education on safe food and water practices. Continuous surveillance of Salmonella susceptibility patterns will not only guide clinicians in selecting appropriate therapy but also support policymakers in developing targeted interventions to reduce disease burden, prevent outbreaks, and curb the spread of antimicrobial resistance. Future studies incorporating molecular resistance mechanisms, population-based surveillance, and treatment outcome data are warranted to better understand the evolving epidemiology of enteric fever and to inform long-term control strategies.
